# Use of Maca Powder (*Lepidium meyenii*) as Feed Additive in Diets of Laying Quails at Different Ages: Its Effect on Performance, Eggshell Quality, Serum, Ileum, and Bone Properties

**DOI:** 10.3390/vetsci9080418

**Published:** 2022-08-08

**Authors:** Esra Tuğçe Gül, Osman Olgun, Alpönder Yıldız, Ahmet Engin Tüzün, Ainhoa Sarmiento-García

**Affiliations:** 1 Department of Animal Science, Faculty of Agriculture, University of Selcuk, Konya 42130, Turkey; 2 Kocarlı Vocational School, University of Aydın Adnan Menderes, Aydın 09970, Turkey; 3 Área de Producción Animal, Department of Construcción y Agronomía, Facultad de Ciencias Agrarias y Ambientales, Universidad de Salamanca, 37007 Salamanca, Spain

**Keywords:** bone traits, egg quality, hormone, intestinal, Maca, performance, quail

## Abstract

**Simple Summary:**

Quail has become a popular poultry species, and it is usually used for egg production in Asia and for meat production in Europe and America. In laying quails, aging negatively affects productive performance, egg parameters, and reproductive hormones, just as it affects their bones and digestive systems. Farmers who raise quails suffer great losses due to this fact. There are several nutrients and bioactive substances in Maca with positive effects on hormones and digestive health, which makes it an excellent option for reducing the effects of aging. This study specifies the benefits of Maca powder on aging quails’ diet. The experimental data indicate that adding 1 g/kg Maca powder to a quail’s diet significantly improved eggshell, ileum, and bone traits that deteriorate with age, without affecting performance. Adding 2 g/kg Maca powder to a quail’s diet also significantly reduced serum cholesterol levels. Incorporating Maca powder into the diet of aged birds could reduce the negative effects of aging.

**Abstract:**

Using additives can reduce the negative effects of aging on factors affecting profitability, such as the availability of nutrients, production, and egg quality. Maca is an herbaceous plant rich in protein, crude oil, essential acids, and pharmacological compounds. Maca has positive effects on different health parameters. In this study, the effect of adding Maca powder to the diets of young and old laying quails at the end of the 10-week trial was investigated. In total, 150 laying Japanese quails (*Coturnix japonica*) (209.1 ± 10.0 g) were randomly distributed to a 2 × 3 factorial arrangement with two ages (10 weeks and 30 weeks) and three Maca powder levels (0, 1, or 2 g/kg), with five subgroups per treatment. According to the study, eggshell quality, total cholesterol, triglyceride, progesterone, and testosterone concentrations of serum were lower in old quail than in young quail, while egg weight, feed intake, and follicle-stimulating hormone increased significantly as quail aged. (*p* < 0.05). Furthermore, aging negatively affected the histomorphology of the ileum and cortical bone thickness (*p* < 0.05). Additional findings show that adding 1 g/kg Maca powder to the diet of quail significantly improved eggshell, ileum, and bone traits that deteriorate with age, without affecting performance, and adding 2 g/kg Maca powder to the diet significantly reduced serum total cholesterol levels (*p* < 0.05). Incorporating Maca powder into the diet of aged birds could reduce the negative effects of aging.

## 1. Introduction

There are many factors such as reproductive hormones, age, stress, and coop conditions that adversely affect performance, egg quality, bone structure, and digestive activities in laying birds [1,2,3]. The age of the bird is one of the most important factors affecting egg production, egg quality, and bird health in the egg industry. With advancing age, the reproductive performance of laying birds begins to decline after the peak laying stage. Animal performance is closely related to gastrointestinal health, including gastrointestinal barrier, digestion, and commensal microbiota [4]. According to the National Research Council [5], with aging, a decrease in performance can be observed in poultry due to the degeneration of features such as villus height, crypt depth, and bacterial composition, which improves the availability of nutrients in the small intestine. In this respect, Liu et al. [6] reported that this decrease could be due to oxidative stress that occurs with egg production over a long time. Age obviously influences both internal and external quality parameters of the eggs. Various changes in the internal composition of the eggs have been described as a consequence of aging [4,7]. Furthermore, aging affects the quantity and quality of collagen fibres, which play an important role in understanding cortical porosity, mineral crystallisation, and bone tissue quality. This fact is a worrying problem for the egg industry, where a 29% prevalence of tibia humerus and keel fractures in end-of-lay caged hen has been described [8]. Therefore, management practices must be employed to enhance the health and egg quality potential of aging laying birds.

*Lepidium meyenii Walp*., more commonly known as “Maca”, is an herbaceous plant of the family Brassicaceae, whose cultivation dates back to the sixteenth century, found in the Andes Mountains in Central Peru, at an altitude of 3700–4500 m [9]. Although the nutrients and metabolites it contains vary according to the regions where it is grown, it is rich in protein, crude oil, essential acids, and minerals [10], as well as pharmacological compounds such as glucosinolate, sterols, fatty acids or their amides (Macamides), alkaloids, and polyphenols [11]. Maca also improves fertility and sexual functions without changing the levels of reproductive hormones [12,13] and regulates health with its anti-osteoporosis and fatigue-reducing properties, among other functions [14]. Furthermore, reports have shown that Maca reduces plasma glucose and fatty acid concentrations [15].

Due to the various health benefits attributed to it, food products derived from Maca have become popular in a niche food market for health-conscious consumers [15]. Researchers have therefore focused on the effects of using a powder or extract of the Maca plant as a food additive for humans [12,13,14]. However, limited studies are available on its use as an ingredient in livestock feed [16,17,18], with most of the available studies involving laboratory rats [19,20,21,22,23,24,25,26]. To our knowledge, there are only two studies examining the effects of adding Maca to quails’ diets [16,27], and only one of them has been carried out on laying quails [16]. Turgud et al. [16] pointed out that the most important positive effects of the study are related to total embryonic death and chick quality characteristics, advising to add 0.1% Maca powder to the diets of breeding flocks. Meanwhile, Olgun et al. [27] showed that the addition of up to 2.0 g/kg Maca powder to growing quail diets could improve feed efficiency, serum testosterone concentration, and ileum properties. In both cases, an improvement of different factors derived from dietary supplementation with Maca powder is suggested. However, due to the scarcity of information pointed out by these authors, the effects of the Maca plant on reproductive and growth traits of Japanese quail should be investigated in future studies.

In light of the above information, the hypothesis of this research is that the administration of Maca in the diet could reduce/eliminate the negative effects observed on performance, digestive system, hormones, and bone traits as a result of aging in quails. For this purpose, the effect of supplementation of Maca powder to the laying quail diet at different ages on performance, egg quality, and serum parameters including hormones, ileum histomorphology, and bone biomechanical traits were evaluated in this research.

## 2. Materials and Methods

### 2.1. Ethical Approval

Criteria specified by European policy for protecting animals were followed during experimental period [28].

### 2.2. Animals and Experimental Diets

A total of 150 laying Japanese quails were randomly allocated to six treatment groups with five replicates, according to a 2 × 3 factorial arrangement design consisting of three levels of Maca powder (0, 1, and 2 g/kg) and two different ages (10 weeks and 30 weeks) in Selçuklu, Konya, and Turkey (38°1′36, 32°30′45). According to the criteria of Narinc et al. [29], young (10 weeks of age; at the weight of 209.1 ± 10.0 g) quails with a peak egg production ranging from 88 to 98% and old (30 weeks of age; at the weight of 238.5 ± 9.0 g) quails were randomly selected for this study.

The basal diet was formulated as isocaloric and isonitrogenic according to the recommendation of the National Research Council [5] for laying quails (Table 1), while the treatment diets were prepared by adding 0, 1, and 2 g/kg Maca powder from a commercial company (Arifoğlu Marketing, Distribution, and Trade Incorporated Company, Istanbul, Turkey) to the control diet, similar to the treatment performed by Turgud et al. [16]. The chemical composition of the experimental diets was analysed according to AOAC [30].

All the birds were fed with a basal diet for two weeks before the study. The quails were housed in identical cages (45 cm × 30 cm) in an environmentally controlled coop (23–25 °C). During the 10-week treatment period, lighting was applied 16 h/day, and water and feed were given *ad libitum*.

### 2.3. Determination of Performance Parameters

Quails were weighed at the beginning and at the end of the experiment with precision weighing balance (±0.01 g). Body weight change was determined by the initial and final weighing (g) of the groups in the trial. The feeds were given to the groups by weighing, and the feed intake was calculated as g/quail/day by subtracting the remaining feed from the total amount at the end of the experiment. Egg production was found as the percentage of the daily collected eggs, and egg weight was obtained by weighing all the eggs collected in the last three days of the treatment. Egg mass was also calculated according to Equation (1).

(1) $$Egg\;mass=\frac{\left(egg\;production\;\left(\%\right)\times egg\;weight\;\left(g\right)\right)}{100}$$

The feed conversion ratio was calculated according to the Equation (2).

(2) $$Feed\;conversion\;ratio=\frac{feed\;intake\;\left(g\;feed\right)\;\;}{egg\;mass\;\left(g\;egg\right)}$$

### 2.4. Determination of Eggshell Quality Parameters

Measurements related to eggshell quality criteria were made for all the eggs collected in the last three days of the experiment (*n* = 300). The specific gravity was identified using a saline solution ranging from 1.080 to 1.090 on the day the eggs were collected according to Holder and Bradford [31]. Eggshell breaking strength was measured using a cantilever system by applying increasing pressure to the broad pole of the shell using the Egg Force Reader (Orka Food Technology Ltd., Ramat Hasharon, Israel). Equation (3) was used to determine the eggshell weight rate.

(3) $$Eggshell\;weight\;rate=\frac{eggshell\;weight\;\left(g\right)\;}{\;egg\;weight\;\left(g\right)}\times 100$$

Membrane eggshell thickness was calculated from the average of the values obtained from the three sections (equator, blunt, and pointed parts) of the eggshells using a digital calliper (Mitutoyo, 0.01 mm, Japan).

### 2.5. Determination of Serum Constituents

Serum constituents were determined according to the modified method described by Long et al. [32]. Blood collection from the quails was carried out between 14:00 and 15:00, and it was collected into coagulation tubes. The serum was isolated by centrifugation at 4000 rpm for 5 min at 4 °C and immediately stored at −20 °C until analysis. The glucose, total cholesterol, triglyceride, total protein, creatinine, calcium, and phosphorus concentrations and follicle-stimulating hormone, luteinising hormone, estradiol, progesterone, and testosterone levels were determined in an auto-analyser using commercial assay kits obtained from Nanjing Jiancheng Bioengineering Institute (Nanjing, China).

### 2.6. Determination of Ileum Histomorphological Parameters

The ileum histomorphological parameters were determined in a total of 30 quails with similar body weight, one from each subgroup, and six measurements were made in each sample. The sample was taken from the ileum as nutrient absorption was almost complete at the terminal end of the small intestine as described by Türgud et al. [33]. The ileum samples taken for histomorphological measurements were immediately buffered in 10% formalin and kept in this solution for 72 h. The intact crypt–villus units of each sample were divided into three cross-sections. Preparation and fixation for the villi and crypts were practiced as Xu et al. [34] reported. Villus height was measured from the crypt–villus junction to the tip of the brush border. Villus width was determined from the midpoint of the villus as much as possible, between the brush borders of the opposing epithelial cells. Crypt depth measurement was taken at the level of the basement membranes of the crypt epithelial cells. The ratio of villus height and crypt depth was calculated dividing *villus height by crypt depth*. Tunica muscularis thickness was defined by measuring the distance, including the lamina muscularis mucosae and excluding the tunica serosa. Villus surface area was calculated with Equation (4) according to Sakamoto et al. [35].

(4) Villus surface area=(2π)×(villus width / 2)×(villus height)

### 2.7. Determination of Bone Biomechanical Properties

One female quail from each subgroup was euthanised by cervical dislocation; the right tibia was taken, and the soft tissues on it were cleaned and stored at −20 °C until measurement (*n* = 30). The samples were kept for 6 h at room temperature and in an air-controlled environment before the evaluation. The mean wall thickness (cortical bone thickness) of the tibia was measured using digital callipers (0.001 mm precision) from two points on the central axis of the fractured tibia, which was used to determine the mechanical properties. The tibia mechanical traits were determined by the load-deformation curve [36] generated using the Universal Testing Machine (Instron 1122) and the Test Works 4 software package (version 4.02; MTS System Corporation, Eden Prairie, MN, USA). The cross-head speed was 5 mm/min. Shear tests on the tibiae were performed using a double-shear block apparatus. Shear force was applied on a 6.35 mm (0.25 inch) section located in the centre of the diaphysis. These tests allowed the evaluation of the ultimate shear force and shear stress for each bone. The cortical bone cross-sectional area was calculated according to Equation (5).

(5) $$cortical\;bone\;cross-sectional\;area=\pi \;\frac{{\left(\frac{\left(\mathrm{diameter}\;\mathrm{of}\;\mathrm{tibia}\;\;\right)}{2}\right)}^{2}-{\left(\frac{\mathrm{cavity}\;\mathrm{diameter}\;\mathrm{of}\;\mathrm{tibia}\;}{2}\right)}^{2}}{2}$$

The shear stress was calculated according to Equation (6).

(6) $$\mathrm{shear}\;\mathrm{stress}=\frac{\mathrm{shear}\;\mathrm{force}}{\mathrm{cortical}\;\mathrm{bone}\;\mathrm{cross}-\mathrm{sectional}\;\mathrm{area}}$$

These biomechanical traits of bone were defined by Wilson and Ruszler [37] and Armstrong et al. [38].

### 2.8. Statistical Analysis

A one-way ANOVA was used to test the effect of the experimental diets on all parameters. If ANOVA showed significant differences among means (main effects), a planned multiple comparison of means was examined by Duncan’s multiple-range test. The statistical differences were defined as *p <* 0.05. All statistical analyses were carried out using the Minitab.

## 3. Results

### 3.1. Performance Parameters

Table 2 shows the effect on performance parameters of different doses of Maca powder in quails of different ages. The results of the statistical analysis with regard to the performance parameters show no significant differences (*p* < 0.05).

Moreover, significant differences were not observed between the old and young groups with regard to egg production, egg mass, and feed conversion rate (*p* < 0.05). However, it was determined that older quails with lower body weight change were also found to have better feed intake and egg weight (*p* < 0.01).

The treatment–age interaction effect was found to be significant in terms of egg weight (*p* < 0.01) and feed intake (*p* < 0.05). The highest egg weight was obtained in the old quails that had no supplementation of Maca to the diet, and the difference between this group and the young group (with and without Maca added to the diet) and the groups of old quails fed with a diet with 1 g/kg Maca powder was found significant (*p* < 0.01). Furthermore, the minimum feed intake was found in the old quails fed with the diet with 1 g/kg Maca powder, and the difference between this group and the old quails fed with 0 and 2 g/kg Maca powder diets was significant (*p* < 0.05).

### 3.2. Eggshell Quality Parameters

The effects of the supplementation of Maca powder in the diets of laying quails at different ages on eggshell quality parameters are shown in Table 3. As shown in Table 3, significant differences were not observed in the damaged eggs, specific gravity, and eggshell weight with or without Maca powder (*p* < 0.05). However, it was determined that adding Maca powder to the diet increased eggshell thickness linearly (*p* < 0.01).

In the current study, statistical differences were determined between the ages in terms of eggshell breaking strength and eggshell thickness. Eggshell breaking strength (*p* < 0.05) and eggshell thickness (*p* < 0.01) decreased as a result of aging in laying quails. The rest of the eggshell quality parameters were not affected by aging (*p* < 0.05). The treatment–age interaction did not show differences for any of the studied parameters (*p* < 0.05).

### 3.3. Serum Biochemical Constituents

Data presented in Table 4 show the effect of Maca powder at different ages on serum biochemical parameters. No effects of Maca powder supplementation on serum biochemical parameters were found (*p* < 0.05). The only exception was the total cholesterol level (*p* < 0.01). This parameter was found to be lower in quails fed with 2 g/kg Maca powder in comparison to the rest of the groups.

Serum total cholesterol and triglyceride levels were affected by age factor. Those values were significantly lower in old quails compared to young ones (*p* < 0.01). The rest of the parameters were not affected by aging (*p* < 0.05).

The treatment–age interaction effect was found to be significant on the total cholesterol. The highest serum total cholesterol level was obtained in the young quails fed with 1 g/kg Maca powder, and the difference between this group and the other groups was significant (*p* < 0.01). In addition, the group that acquired the minimum serum total cholesterol level among the interactions was the old quails group fed with a diet supplemented with 1 g/kg Maca powder, and no significant diversity among the interaction groups consisting of old quails was observed (*p* < 0.05).

### 3.4. Serum Hormone Concentrations

Table 5 shows the effects of age, the administration of Maca powder, and their interactions on serum hormone concentrations. Significant differences were not observed (*p* < 0.05) between the test and control groups with regard to the serum hormone concentrations. The addition of Maca powder only affected serum testosterone concentration (*p* < 0.05). There was a significant increase in the testosterone concentration with administration of 2 g/kg Maca powder compared to the rest of the experimental groups.

No statistical differences were found in serum-luteinising hormone (LH) and estradiol between quails of different ages (*p* < 0.05). However, the serum follicle-stimulating hormone (FSH) level was higher in old quails compared to young quails (*p* < 0.01), while the serum progesterone (*p* < 0.05) and testosterone (*p* < 0.01) levels were lower.

The treatment–age interaction effect was found to be significant on the serum level of the luteinizing hormone (*p* < 0.01) and testosterone level (*p* < 0.05). The groups showing the minimum and maximum results were young quails fed with 1 and 2 g/kg Maca powder, respectively, and the difference between these two groups was statistically significant. Meanwhile, the group with the lowest testosterone level was the old quails whose diets did not include Maca, and the difference between this group and the other groups, except the group of old quails whose diet contained 1 g/kg Maca, was statistically significant.

### 3.5. Ileum Histomorphological Parameters

The effects of the addition of Maca powder to the diets of laying quails at different ages on the ileum histomorphology are shown in Table 6. The addition of Maca powder to laying quail diets increased the villus width of the ileum, whereas the villus height decreased (*p* < 0.001). Furthermore, differences were found in crypt depth and villus height/crypt depth due to Maca powder supplementation (*p* < 0.001). The results reveal that quails in the 1 g/kg Maca group achieved the lowest crypt depth but the highest villus/crypt height compared to the rest of the experimental groups.

Aging affected all ileum histomorphological parameters, except the crypt depth of ileum (*p* < 0.05). A significant decrease was observed in villus width, villus height, villus height/crypt depth, and villus surface area as a result of quail aging (*p* < 0.001).

Villus width, villus height, villus height/crypt depth, and villus surface area were significantly affected by age × Maca powder interactions (*p* < 0.01). When the effect of such interactions on ileum histomorphology was examined, the villus width was found to have significantly increased with the addition of 1 and 2 g/kg to young quails’ diets compared to those of the other groups (*p* < 0.01). The maximum villus height in the interaction groups was obtained in the young quail group without Maca powder added to their diet, and the difference between this group and the other groups was statistically significant (*p* < 0.01). The highest crypt depth among the interaction groups was obtained with the addition of 2 g/kg Maca powder to the diets of old quails, and the difference between this group and all the other groups (except the young quail group without Maca in their diet) was found to be considerably significant (*p* < 0.01). The villus surface area was significantly affected by age × Maca powder interactions (*p* < 0.01), and the highest numerical value was obtained in young quails with 1 g/kg Maca added to their diets; the difference between this group and all the old quail groups was also statistically significant.

### 3.6. Bone Biomechanical Properties

As shown in Table 7, significant differences were not observed (*p* < 0.05) in the bone biomechanical properties with or without Maca powder of the laying quails throughout the current study. Moreover, significant differences were not observed between the young and old quails with regard to the bone biomechanical properties except for cortical bone thickness. Cortical bone thickness was affected by age, as it was lower in old quails compared to the young quails (*p* < 0.05). The treatment–age interaction did not show differences for any of studied parameters (*p* < 0.05); however, a trend was observed for cortical bone cross sectional area (*p* = 0.071).

## 4. Discussion

### 4.1. Performance Parameters

In the current study, it was determined that the addition of Maca powder to diet had no effect on performance parameters. The results of this study are consistent with the results reported by Turgud and Narinç [16], Korkmaz et al. [39], and El-Sheikh et al. [17] in Japanese quails, broiler chickens, and rabbits respectively. At the same time, Olgun et al. [27] also stated that feed intake did not significantly change with the addition of Maca powder to the diet of growing quails. It has also been reported that Maca powder supplementation did not affect growth rate or feed intake in rats [23,40]. Similar results were reported by Staerfl et al. [18] in bulls, where Maca powder did not affect the feed intake or growth rate at the end of a 9-month experiment which is consistent with the results obtained by Clément et al. [41]. However, studies on juvenile fish and alevins found that Maca root meal significantly improved performance traits such as feed intake, feed conversion, growth rate, and protein efficiency ratio [42]. In another study, Wan et al. [43] examined the effects of different levels of Maca aqueous extract (300, 600, and 1200 mg/kg) on golden hamsters fed a high-fructose diet and concluded that body weight gain was greatly reduced at 1200 mg/kg, while feed efficiency improved at 300 and 1200 mg/kg. As Beharry and Henrich [44] asserted, the effectiveness of Maca powder or extract on performance depends on factors including animal species, physiological biomarkers, and experimental period, which is to say the susceptibility of the chosen trial model system.

In the current study, the age factor decreased body weight change, while it increased egg weight and feed intake. Similar to those results, Nazligül et al. [45] and Orhan and Aktan [46] reported that the egg weight increased continuously with aging in laying quails. On the other hand, Zita et al. [47] noted that the highest egg weight was achieved in quails at 25 weeks of age and gradually decreased until the age of 49 weeks. Meanwhile, Carneiro et al. [48] explained that egg weight was not affected by the age factor in their study with quails aged 80, 160, 240, and 390 days. In this research, the increase in feed intake can be connected with the advance in egg weight.

Moreover, the highest feed intake was recorded in the old group with 2 mg/kg Maca powder, which is consistent with the results obtained by Turgud and Narinç [16]. Those authors showed that the effect of adding Maca powder to the feed intake increases linearly with age and is thought to have appetizing properties.

### 4.2. Eggshell Quality Parameters

According to these results, Maca powder had neither positive nor adverse impacts on eggshell quality parameters, except on eggshell thickness. The addition of Maca powder to the diet increased eggshell thickness, but this effect occurred without affecting serum calcium and phosphorus levels, as shown in Table 4. This effect could be due to the improvement of the intestinal absorption of nutrients and their transport to the egg in the isthmus. Studies investigating the effects of Maca powder on egg quality parameters are scarce. The only study found in the literature on the aforementioned parameters [39] reported that eggshell breaking strength and eggshell thickness were not affected by the treatments when different levels (5 and 10 g/kg) of Maca powder were added to the diets of laying hens, which is consistent with the results obtained in this study.

As expected, eggshell thickness and eggshell weight decreased with aging, which is consistent with the findings of previous authors such Nazligül et al. [45], Orhan and Aktan [46], and Zita et al. [47]. The most important reason for eggshell quality to be negatively affected by aging could be changes in nutrient absorption [49]. Intestinal activity dysfunction with aging could decrease the bioavailability of calcium [50], and this causes the inhibition of ion conduction and endometrial formation, leading to the degradation of the structural properties of the eggshell [51]. With aging, the palisade layer, which is one of the layers that forms the eggshell and gives the egg its resistance to breakage, becomes thinner, leading to a weakening of the eggshell [51,52]. In addition, the decimated eggshell breaking strength caused by aging can be associated with an increase in egg weight [4,53], as shown in this study. However, contrary to the current results, Carneiro et al. [48] reported that eggshell thickness was not affected by age factor in their study with 80, 160, 240, and 390-day-old quails. Meanwhile, Nagarajan et al. [54] announced that no relationship existed between eggshell thickness and age in quails. Differences between previous studies could be due to the age of the quails used in the experiment and the duration of the experiment.

### 4.3. Serum Biochemical Constituents

The addition of Maca powder to the diet only affected the serum total cholesterol level, which was reduced with the addition of 2 g/kg Maca powder to the diet. The results of this study are consistent with the results reported by Olgun et al. [27] for growing quails. In another study conducted in golden hamsters, it was reported that the addition of aqueous Maca extract to the diet (300, 600, or 1200 mg/kg) decreased levels of total cholesterol but increased triglyceride concentration of serum, except for 300 mg/kg. Similar results have been found for experiments on rats [55]. Researchers reported a reduction in blood glucose, plasma and liver cholesterol, and triglyceride levels when 10 g of Maca powder was added to the diets of rats and mice with hereditary hypertriglyceridaemia who consumed high levels of sucrose. Maca could be used to prevent and treat chronic diseases, according to researchers. Additionally, Zhang et al. [40] clarified that adding Maca ethanol extract to the diets of rats at 0.5 and 1.25 g/kg decreased serum cholesterol and also reported that Maca was effective in regulating lipid concentration, but which active ingredient this effect is related to was not clear. On the other hand, Korkmaz et al. [39] stated that 5 and 10 g/kg of Maca powder added to laying hens’ diet did not significantly affect serum cholesterol and triglyceride concentrations. Similar, Meissner et al. [56] stated that 0.75 or 7.5 g of gelatinised Maca powder added to the diet did not affect serum cholesterol and triglyceride levels in rats.

In the study, it was determined that the age factor influenced the total cholesterol and triglyceride of quails. Generally, the fact that most of the serum parameters reflect physiological, nutritional, and even pathological changes in the body signifies that they are important indicators of the status of health in the body [57]. These biochemical indicators are closely related to homeostasis [58]. Serum total cholesterol and triglyceride levels were significantly lower in old quails compared to young ones. Similarly, Suchy et al. [59] remarked that plasma cholesterol levels decreased late in hens during the 5th and 50th weeks of the laying period. In contrast, Kraus et al. [60] noted that the age factor did not affect serum cholesterol and triglyceride concentrations in 34-, 42-, and 50-week-old local laying hens. Cerolini et al. [61], who determined that there were age-related variations in the haematological indicators, explained that there were significant changes in plasma cholesterol and triglyceride levels with aging and reported that the lowest plasma lipid level was observed in young hens (18 weeks) and that the concentration increased as a result of aging (from 18 to 67 weeks).

### 4.4. Serum Hormone Concentrations

It was determined that the addition of Maca powder to quail diets did not cause any difference in terms of serum hormone concentrations except for testosterone levels. The addition of 2 g/kg Maca powder to the diet increased serum testosterone concentration in quails. Similar results were reported by Olgun et al. [27] in male growing quails. However, those authors did not found differences for any serum hormone concentration in female growing quails. Having obtained similar results, Meissner et al. [56] expressed that the supplementation of 0.75 or 7.5 g of gelatinised Maca did not affect the FSH, the LH, and estradiol but increased the progesterone level and stated that this increase could be due to the complex phytochemicals contained in Maca. In another study conducted in rats, Gonzales et al. [24] reported a higher serum testosterone level in the group treated with yellow Maca, which is consistent with the results obtained by Onaolapo et al. [62] and Oshima et al. [63] in mice. This increase was due to the synergistic effect of the saponin, arginine, lead, and vitamin E contained in Maca. Research by Ohta et al. [64] showed similar trends. The experiment was conducted in young male Wistar rats fed a hydroalcoholic extract of Maca powder (a combination of yellow, black, and red Maca) for 6 weeks. This study shows that the administration of a hydroalcoholic extract of Maca for 42 days raised serum testosterone levels. These findings suggest that Maca can stimulate Leydig cells, especially in the metabolic process of cholesterol, as shown in the current experiment. On the other hand, Zhang et al. [40] stated that in their study conducted with ovariectomised rats, Maca ethanol extract at the levels of 0.5 and 1.25 g/kg reduced the follicle-stimulating hormone, but testosterone concentration was not affected by the treatments. Study results on men show that the gelatinized form containing 1.75 g daily [65] and the 1.5 or 3 g/day Maca powder [66] did not influence follicle-stimulating hormone, luteinizing hormone, and testosterone levels. Similar, another study found that testosterone levels were not affected by red Maca in adult rats [67]. As is known, Maca presents different ecotypes in relation to the different colours of its hypocotyls. Furthermore, different biological properties have been attributed to different colours. According to Tafuri et al. [68], the differences between the above results could be justified for this reason.

The performance traits of laying birds are affected by reproductive hormones, lipid and mineral metabolism, aging, and stress. In the current study, FSH concentration increases with aging, while levels of progesterone and testosterone are inversely correlated with aging. The findings of the current study disagree with the findings of Petritz et al. [69], who reported that the progesterone level of serum in quails did not change significantly up to 26 weeks of age. In another study conducted in laying hens, meanwhile, Braw-Tal et al. [70] determined that plasma progesterone concentration did not change from 245 to 700 days of age but decreased at 800 days of age. Schreiweis et al. [71] observed that aging reduces estrogen synthesis by the ovaries and aged hens have low serum estrogen levels.

### 4.5. Ileum Histomorphological Parameters

Both the villus and the crypts play an important role in the small intestine’s absorption [72]. A dynamic balance in the regeneration of the intestinal epithelium is reflected in the production of enterocytes and desquamation in the crypts [73]. In the current study, it was determined that the addition of Maca powder to the laying quails’ rations improved villus width. However, the effect of Maca powder on the other intestinal parameters tested is controversial and requires further research. To our knowledge, there is only one study that has examined the effects of Maca powder on the histomorphological features of the small intestine in quails [27]. These authors stated an improvement in all histomorphological parameters of the small intestine with the addition of Maca powder to the diet [27], which is partially in agreement with our findings. In agreement with these results, Canales et al. [74] proposed that Maca could be used in malabsorption syndrome, given its properties that can improve digestion. Medicinal plants that contain phenolic and bioactive components similar to Maca have shown positive effects on the digestive system. Su et al. [73] stated that the mixture of essential oils of certain plants did not affect the villus height of broilers but decreased crypt depth as the inclusion of this ingredient increased. Similar findings were reported by Hafeez et al. [75], who stated that coconut oil improves the integrity of the intestinal wall. The improved intestinal dimensions may also be due to the antioxidant effect of essential oils.

This study shows that villus width, villus height, villus height/crypt depth, and villus surface area decreased linearly as age increased. With aging in animals, the functions of the organs that play a role in the digestive system tend to decrease, which causes the digestion of nutrients to be limited [76]. In accordance with the results of this study, Gu et al. [7] reported that the villus height and villus height/crypt depth of hens aged 525 days were significantly higher than those of hens aged 195 days.

The highest crypt depth among the interaction groups was obtained with the addition of 2 g/kg Maca powder to the diets of old quails. A Maca supplementation offers an interesting alternative to slow down the effects of aging at the intestinal level.

### 4.6. Bone Biomechanical Properties

The results obtained in this study show that the addition of Maca to the quails’ diets had a positive effect on the shear force of bones, which is consistent with the findings of Olgun et al. [27]. Zhang et al. [40] clarified that 0.24 g/kg ethanol extract of Maca powder increased the thickness of the femoral diameter in ovariectomised rats. Gonzales et al. [77] explained that 63 mg/mL of Maca hydraulic extract added to the diet of ovariectomised rats was effective in protecting their bone structure and referred to Macaene, Macamides, polyphenols, polyunsaturated fatty acids, and phytosterols as the reason for this effect. Although studies are limited in animals, it has been observed that in women, red Maca powder is known to be the most effective type for its hormone-balancing effects and its action on bone health. Researchers who noted that Maca powder was an effective herbal feed additive to prevent osteoporosis stated that the active ingredients were calcium, magnesium, and phytochemicals [40,55].

In this study, cortical bone thickness significantly decreased with aging, which is consistent with those results obtained by Regmi et al. [8] in laying hens’ cortical bones. In the same study, the cortical bone cross-sectional area was reduced as a result of aging. Similar findings were reported by [71]. With age, bone strength decreases due to the high demand for calcium from bone for egg formation, which is accelerated by caged birds’ inactivity [52]. As shown in this study, laying animals lose structural bone during the late laying period, causing serious skeletal conditions such as osteoporosis, affecting animal health and welfare, and resulting in economic losses. Osteoporosis refers to structural bone loss that increases the risk of fractures [78]. A trend was observed (*p* = 0.071) for cortical bone cross-sectional area. The highest value was observed for young quails that had received 1 g/kg Maca powder, followed by old quails that had received 2 g/kg Maca powder. According to these findings, the inclusion of Maca powder in the diet of older quail could reduce bone damage caused by aging.

## 5. Conclusions

According to this research, the addition of Maca powder to the diet of laying quails resulted in an increase in eggshell thickness, serum testosterone concentration, tibia shear force, and villus width, along with a decrease in villus height and serum total cholesterol level. The addition of 1 g/kg Maca powder to laying quails’ diets improved eggshell quality, bone, and small intestine parameters without affecting performance, while 2 g/kg Maca powder was effective in reducing serum cholesterol.

The results obtained in this study show that quail aging leads to an increase in egg weight, feed intake, and serum follicle-stimulating hormone level as well as a decrease in eggshell quality, total cholesterol, triglyceride, progesterone, and testosterone concentrations of serum, tibia cortical bone thickness, and ileum health.

## Figures and Tables

**Table 1:** Ingredients and nutrient contents of the diet.

Ingredients	%	Nutrient Contents	%
Corn	53.20	Metabolizable energy, kcal ME/kg	2902
Soybean meal	28.70	Crude protein	20.01
Sunflower meal	4.00	Calcium	2.50
Wheat bran	2.00	Available phosphorus	0.35
Sunflower oil	4.60	Lysine	1.01
Limestone	5.60	Methionine	0.45
Dicalcium phosphate	1.14	Methionine + cystine	0.82
Salt	0.35		
Premix ^1^	0.25		
DL methionine	0.16		
Total	100.00		

^1^ Supplied per kg diet: Trans-retinol: 3.6 mg, Cholecalciferol: 0.1 mg, Menadione: 5 mg, α-tocopherol acetate: 75 mg, Thiamine: 3 mg, Riboflavin: 6 mg, Pyridoxine: 5 mg, Cyanocobalamin: 0.03 mg, Nicotinic acid: 40 mg, Pantothenic acid: 10 mg, Folic acid: 0.75 mg, D-biotin: 0.075 mg, Choline chloride: 375 mg, Manganese: 80 mg, Iron: 60 mg, Zinc: 60 mg, Copper: 5 mg, Selenium: 0.15 mg, Cobalt: 0.20 mg, Iodine: 1 mg.

**Table 2:** Effects of Maca powder supplementation in diets of laying quails at different ages on performance parameters.

Age	Maca Powder, g/kg	Body Weight Change (g)	Egg Production (%)	Egg Weight (g)	Egg Mass (g/d/Quail)	Feed Intake (g/d/Quail)	Feed Conversion Rate (g Feed/g Egg)
Young		27.17 ^a^	83.58	12.55 ^b^	10.50	29.33 ^b^	2.81
Old		3.72 ^b^	85.41	13.05 ^a^	11.14	31.03 ^a^	2.80
SEM ^1^		3.24	2.015	0.115	0.286	0.445	0.076
	0	18.38	84.37	12.90	10.87	30.84	2.86
	1	10.82	83.17	12.65	10.53	29.53	2.83
	2	17.15	85.95	12.85	11.06	30.17	2.74
	SEM ^1^	5.87	2.504	0.168	0.370	0.559	0.092
Young	0	30.00	86.39	12.35 ^c^	10.67	30.10 ^a,b,c^	2.84
Young	1	24.71	82.34	12.72 ^b,c^	10.49	29.60 ^b,c^	2.86
Young	2	26.81	82.01	12.60 ^b,c^	10.34	28.30 ^c^	2.75
Old	0	6.75	82.34	13.46 ^a^	11.08	31.58 ^a,b^	2.88
Old	1	−3.06	84.00	12.58 ^b,c^	10.58	29.46 ^c^	2.80
Old	2	7.48	89.89	13.11 ^a,b^	11.77	32.05 ^a^	2.73
SEM ^1^		5.30	3.439	0.149	0.491	0.621	0.137
*p*-Value							
Age		0.001	0.535	0.002	0.139	0.005	0.916
Maca		0.399	0.736	0.273	0.587	0.160	0.690
Age × Maca		0.772	0.267	0.005	0.398	0.026	0.934

^1^ Standard error means. ^a–c^ Values within a row with different superscripts differ significantly at *p* < 0.05.

**Table 3:** Effects of Maca powder supplementation in diets of laying quails at different ages on eggshell quality parameters.

Age	Maca Powder, g/kg	Damaged Eggs (%)	Specific Gravity (g/cm^3^)	Eggshell Breaking Strength (kg)	Eggshell Weight (%)	Eggshell Thickness (µm)
Young		2.21	1.073	1.45 ^a^	8.10	154.8 ^a^
Old		0.85	1.072	1.36 ^b^	8.11	137.7 ^b^
SEM ^1^		0.697	0.001	0.032	0.107	2.71
	0	1.26	1.073	1.35	8.10	136.6 ^c^
	1	1.79	1.071	1.41	8.02	147.3 ^b^
	2	1.53	1.074	1.46	8.19	154.9 ^a^
	SEM ^1^	0.912	0.001	0.040	0.130	3.72
Young	0	1.12	1.073	1.43	8.07	143.3
Young	1	3.03	1.071	1.38	7.98	156.7
Young	2	2.47	1.075	1.53	8.24	164.3
Old	0	1.40	1.072	1.26	8.14	129.9
Old	1	0.55	1.071	1.43	8.06	137.9
Old	2	0.58	1.073	1.39	8.13	145.5
SEM ^1^		1.096	0.001	0.046	0.197	2.49
*p*-Value						
Age		0.250	0.427	0.044	0.943	0.001
Maca		0.932	0.166	0.103	0.705	0.001
Age × Maca		0.595	0.986	0.071	0.858	0.545

^1^ Standard error means. ^a–c^ Values within a row with different superscripts differ significantly at *p* < 0.05.

**Table 4:** Effects of Maca powder supplementation in diets of laying quails at different ages on serum biochemical properties.

Age	Maca Powder, g/kg	Glucose (mg/dL)	Total Cholesterol (mg/dL)	Triglyceride (mg/dL)	Total Protein (g/dL)	Creatinine (mg/dL)	Calcium (mg/dL)	Phosphorus (mg/dL)
Young		293	179.3 ^a^	837 ^a^	4.48	0.331	22.63	5.99
Old		303	135.8 ^b^	675 ^b^	4.51	0.343	23.58	5.83
SEM ^1^		5.7	9.52	29.6	0.248	0.007	1.318	0.367
	0	293	161.8 ^a^	800	4.28	0.333	22.21	5.35
	1	294	173.6 ^a^	698	4.95	0.333	24.31	6.50
	2	308	137.3 ^b^	770	4.25	0.346	22.80	5.89
	SEM ^1^	6.5	11.66	44.0	0.243	0.009	1.579	0.433
Young	0	287	172.3 ^b^	877	4.08	0.325	21.23	5.83
Young	1	286	226.3 ^a^	747	5.30	0.333	24.23	6.38
Young	2	306	139.3 ^b,c^	886	4.05	0.335	22.45	5.78
Old	0	299	151.3 ^b,c^	723	4.48	0.340	23.20	4.88
Old	1	301	121.0 ^c^	649	4.60	0.333	24.40	6.63
Old	2	310	135.3 ^b,c^	654	4.45	0.358	23.15	6.00
SEM ^1^		8.8	10.32	47.19	0.328	0.012	2.314	0.563
*p*-Value								
Age		0.225	0.001	0.001	0.922	0.236	0.642	0.778
Maca		0.283	0.014	0.139	0.182	0.461	0.685	0.263
Age × Maca		0.876	0.001	0.418	0.325	0.663	0.932	0.608

^1^ Standard error means. ^a–c^ Values within a row with different superscripts differ significantly at *p* < 0.05.

**Table 5:** Effects of Maca powder supplementation in diets of laying quails at different ages serum hormone concentrations.

Age	Maca Powder, g/kg	Follicle-Stimulating Hormone (IU/L)	Luteinizing Hormone (IU/L)	Estradiol (pg/mL)	Progesterone (ng/mL)	Testosterone (ng/dL)
Young		0.132 ^b^	0.238	58.13	1.321 ^a^	28.04 ^a^
Old		0.203 ^a^	0.241	55.12	0.959 ^b^	21.33 ^b^
SEM ^1^		0.015	0.008	5.383	0.108	1.655
	0	0.186	0.235	59.10	1.333	23.56 ^b^
	1	0.179	0.238	51.32	1.055	21.97 ^b^
	2	0.136	0.246	59.45	1.033	28.53 ^a^
	SEM ^1^	0.021	0.009	6.553	0.141	1.999
Young	0	0.173	0.235 ^a,b^	59.62	1.658	30.75 ^a^
Young	1	0.120	0.215 ^b^	48.09	1.243	23.94 ^a,b^
Young	2	0.103	0.265 ^a^	66.68	1.063	29.44 ^a^
Old	0	0.200	0.235 ^a,b^	58.58	1.008	16.38 ^c^
Old	1	0.238	0.260 ^a,b^	54.54	0.868	20.00 ^b,c^
Old	2	0.170	0.228 ^a,b^	52.23	1.003	27.62 ^a^
SEM ^1^		0.020	0.010	9.158	0.173	2.159
*p*-Value						
Age		0.002	0.781	0.712	0.023	0.002
Maca		0.119	0.563	0.653	0.203	0.025
Age × Maca		0.215	0.005	0.570	0.281	0.028

^1^ Standard error means. ^a–c^ Values within a row with different superscripts differ significantly at *p* < 0.05.

**Table 6:** Effects of Maca powder supplementation in diets of laying quails at different ages on ileum histomorphological parameters.

Age	Maca Powder, g/kg	Villus Width (µm)	Villus Height (µm)	Crypt Depth (µm)	Villus Height/Crypt Depth	Villus Surface Area (mm^2^)
Young		96 ^a^	556 ^a^	73	7.85 ^a^	0.167 ^a^
Old		88 ^b^	501 ^b^	72	7.28 ^b^	0.139 ^b^
SEM ^1^		1.0	7.0	1.3	0.100	0.003
	0	86 ^b^	554 ^a^	77 ^a^	7.39 ^b^	0.149
	1	94 ^a^	503 ^c^	65 ^b^	7.98 ^a^	0.150
	2	95 ^a^	530 ^b^	77 ^a^	7.18 ^b^	0.158
	SEM ^1^	1.3	8.8	1.5	0.147	0.003
Young	0	87 ^b^	597 ^a^	79 ^a,b^	7.75 ^a,b^	0.161 ^a,b^
Young	1	101 ^a^	541 ^b^	71 ^c^	7.95 ^a^	0.174 ^a^
Young	2	98 ^a^	534 ^b^	70 ^c^	7.84 ^a,b^	0.164 ^a,b^
Old	0	86 ^b^	513 ^b,c^	75 ^b,c^	7.04 ^b,c^	0.137 ^c^
Old	1	88 ^b^	473 ^c^	61 ^d^	8.01 ^a^	0.132 ^c^
Old	2	91 ^b^	526 ^b^	83 ^a^	6.51 ^c^	0.151 ^b^
SEM ^1^		1.7	11.6	2.0	0.201	0.005
*p*-Value						
Age		<0.001	<0.001	0.971	<0.001	<0.001
Maca		<0.001	<0.001	<0.001	<0.001	0.225
Age × Maca		0.003	0.004	<0.001	0.002	0.009

^1^ Standard error means. ^a–c^ Values within a row with different superscripts differ significantly at *p* < 0.05.

**Table 7:** Effects of Maca powder supplementation in diets of laying quails at different ages on bone biomechanical properties.

Age	Maca Powder, g/kg	Cortical Bone Thickness (mm)	Cortical Bone Cross-Sectional Area (mm^2^)	Shear Force (n)	Shear Stress (N/mm^2^)
Young		0.468 ^a^	1.829	197	110.5
Old		0.395 ^b^	1.688	193	117.4
SEM ^1^		0.023	0.099	8.1	7.10
	0	0.408	1.709	173 ^b^	103.6
	1	0.442	1.755	200 ^a^	119.5
	2	0.444	1.813	212 ^a^	118.8
	SEM ^1^	0.029	0.123	7.7	8.18
Young	0	0.446	1.830	167	93.6
Young	1	0.515	1.998	212	109.8
Young	2	0.441	1.660	212	128.1
Old	0	0.369	1.588	179	113.5
Old	1	0.369	1.513	188	129.2
Old	2	0.446	1.965	212	109.6
SEM ^1^		0.034	0.152	10.7	10.62
*p*-Value					
Age		0.034	0.304	0.675	0.485
Maca		0.585	0.818	0.011	0.339
Age × Maca		0.178	0.071	0.318	0.209

^1^ Standard error means. ^a,b^ Values within a row with different superscripts differ significantly at *p* < 0.05.

## Data Availability

The data analyzed for the study are available from the corresponding author upon reasonable request.
